# Exploring Eating Habits, Healthy Food Awareness, and Inclination toward Functional Foods of Italian Elderly People through Computer-Assisted Telephone Interviews (CATIs)

**DOI:** 10.3390/nu16060762

**Published:** 2024-03-07

**Authors:** Giulia Maria Daniele, Chiara Medoro, Nico Lippi, Marta Cianciabella, Massimiliano Magli, Stefano Predieri, Giuseppe Versari, Roberto Volpe, Edoardo Gatti

**Affiliations:** 1 Institute for BioEconomy, National Research Council (CNR), Via Piero Gobetti 101, 40129 Bologna, Italy; giuliamaria.daniele@ibe.cnr.it (G.M.D.); nico.lippi@ibe.cnr.it (N.L.); marta.cianciabella@ibe.cnr.it (M.C.); massimiliano.magli@ibe.cnr.it (M.M.); stefano.predieri@ibe.cnr.it (S.P.); giuseppe.versari@ibe.cnr.it (G.V.); edoardo.gatti@ibe.cnr.it (E.G.); 2 Health and Safety Unit (SPP), National Research Council (CNR), Piazzale Aldo Moro, 7, 00185 Roma, Italy; roberto.volpe@cnr.it

**Keywords:** senior consumers, food co-creation, food preferences, diet, health enhancing factors

## Abstract

The continuous increase in life expectancy leads to progressive population ageing, especially in most developed countries. A healthy diet and better consumption of tailored functional foods may represent one of the strategies to postpone or slow down age-related decrements, thus increasing healthy ageing and reducing healthcare costs. This research aimed to explore elderly people’s (>65 years old) eating habits and assess their awareness of food-health correlation. In total, 511 Italian seniors answered a CATI (computer-assisted telephone interviewing) questionnaire through a deep, telephone interview to collect information about dietary habits, healthy food awareness, and inclination for functional foods. The elderly were divided into four groups according to gender and age: Early Elderly Female (*n* = 130), Early Elderly Men (*n* = 109), Late Elderly Female (*n* = 157), and Late Elderly Men (*n* = 115). The groups provided a positive self-assessment of health status and individual diet healthiness, which were both considered over “good enough” (5 on 10-point scale) and showed food consumption habits in line with the Mediterranean Diet (MD) principles. The daily diet was based on fruits, vegetables, bread, and pasta, with extra virgin olive oil as the main fat source, all over “often” consumed (4 on 5-point scale). Old people also showed awareness of different food’s healthy properties. Specifically, females were more aware of food’s impact on health, considered close to “extremely healthy” (9 on 10-point scale), and strictly followed a MD. Participants also expressed optimistic expectations about functional food efficiency, evaluated as close to “extremely desirable” (8 or 9 on 10-point scale), against age-related problems, highlighting the most important as diabetes, overweight, intestine problems, and low mood. The interviewed elderly were also involved in virtual functional food co-creation, indicating through a basic matrix which, among the most familiar foods, could be the ideal functional food, focusing on fruitsand vegetables. A pleasant odor/flavor, a liquid texture, and a warm serving temperature rather than cold characterized the virtual functional food created. Other positive attributes were liquid and thickness, while acidity and bitterness were among the least desired traits. These findings show how elderly people, despite predictable age-related sensory and cognitive loss, when properly involved and guided, can help envision foods that fit their needs and desires.

## 1. Introduction

The population’s average lifespan is increasing in large parts of certain countries, especially the most developed ones. In the last twenty years, the elderly Italian population (>65 years old) has significantly increased. In the year 2000, the over 60 amounted to 10.3 million, and by the first of January 2019 had reached 13.8 million, accounting for 22.8% of the total population [1]. Today, the life expectancy at birth in Italy is over 80 for men and over 85 for women [1]. However, there is a high risk that the years of life earned might be characterized by sickness and disabilities. Ageing is influenced by both hereditary traits and environmental factors such as poor nutrition, bad lifestyle, pollution, and stress. Thus, specific approaches to postpone or slow down age-related decrements in cognition and mobility are necessary to increase healthy ageing and reduce healthcare costs [2]. Indeed, most of these degenerative diseases, as highlighted by several studies, can be prevented through focused physical activity and a balanced diet [1,3,4]. Moreover, cardiovascular diseases, cancer, and dementia, the most common age-related conditions, are increasing due to improper nutrition [5].

The positive impact of healthy food habits can be boosted through the consumption of functional foods (FFs), which provide specific healthy benefits in addition to their basic nutritional value [6], helping reduce or delay the risk of diseases [7], especially if tailor-made for the elderly’s needs. Indeed, FFs have usually been developed to prevent and cure some of the most common chronic disorders in vulnerable people, such as the elderly [8], since they provide nutrients which improve biological activities and ensure physiological benefits, reducing the rate risk of chronic age-related diseases. Among others, FFs boost the immune system, increase energy, maintain healthy skin, joints and bones, and improve digestion and metabolism, fostering longevity [9]. 

The economic interest in FFs is growing, as shown by the increasing number of FFs available on the market, highlighting a strong focus on diet-related diseases prevention. Thus, many studies have investigated consumer attitudes and motivations toward FFs [10], as well as their beneficial effects in old adults. Rudkowska et al., 2010 [11], have shown that phytosterol-rich FFs are effective against LDL cholesterol, while Rampelli et al., 2013 [12], have discovered that FFs rich in probiotics boost the gut microbiota.

This study, within the Italian research project “Nutrage”, investigated eating behaviors, knowledge about food benefits, and inclination toward the consumption of FFs as strategies against age-related disorders in a population of old Italian adults. Moreover, the use of computer-assisted telephone interviewing (CATI), a deep interview method used in social sciences and health care [13], as well as a co-creation task to set the ideal FF, were tested within an old consumer population.

## 2. Materials and Methods

### 2.1. Study Design

A cross-sectional survey was conducted in October 2020. Italian elderly people were recruited and interviewed through using the CATI method (Computer-Assisted Telephone Interviewing), which is particularly suitable for old consumers [14,15]. The interviews lasted 20 min. The questionnaire was developed through a multidisciplinary approach by researchers from food, consumer science, and medical departments.

All the participants answered the questionnaire voluntarily; they were informed of the main research outcomes and gave consent for their data to be used. The study was conducted in accordance with the Declaration of Helsinki, and the right to privacy and data protection was respected following current legislation (GDPR 2016/679).

### 2.2. Deep Interview Questionnaire

The questionnaire was structured in five sections.

#### 2.2.1. Socio-Demographic and Health Status Self-Assessment 

Non-institutionalized Italian older adults, aged between 65 and over, were interviewed by telephone. The participants were requested to indicate their gender, geographical area of origin, education level, and NUTS region of residence (north-east, north-west, center, south, and islands). Participants were also screened based on their age class: Early Elderly Female (EEF), Early Elderly Male (EEM), Late Elderly Female (LEF) and Late Elderly Male (LEM) [16]. 

Health status self-assessment was requested to better define cohort features: (I) actual individual health level (1: not good at all; 5: good enough; 10: absolutely good); (II) diet healthiness (1: not healthy at all; 5: healthy enough; 10: absolutely healthy), and (III) estimation of physical activity consistency (1: not at all; 5: regular enough; 10: very regular).

#### 2.2.2. Foods Consumption Frequency, Healthiness Assessment of Foods, and Bioactive Food Components

Consumers were then asked to indicate the consumption frequency of 17 Italian common foods (fruit, vegetables, legumes, bread, pasta, cheese, red and processed meat, white meat, eggs, wine, extra virgin olive oil, butter, sweets, tea, yogurt, seafood, and spices) using a 5-point scale (1: “never”; 2: “rarely”; 3: “sometimes”; 4: “often”; 5: “everyday”). For each food type, the participants were also requested to indicate their healthiness using a 10-point scale (from 1: “not healthy at all” to 10: “very healthy”). In addition, they were presented with a list of 9 bioactive food components (calcium, sodium, iodine, potassium, proteins, fibers, antioxidants, prebiotics, probiotics) and requested to indicate if they are supposed to have a positive (Yes) or negative (No) effect or if they were not aware of any function (I do not know).

#### 2.2.3. Expectations about Functional Foods Healthy Benefits

In this section, elderly awareness of FFs’ healthy benefits was explored.

Consumers, were first informed on the definition of FFs as proposed by Temple, 2022 [17]: “Functional foods are novel foods that have been formulated so that they contain substances or live microorganisms that have a possible health-enhancing or disease-preventing value, and at a concentration that is both safe and sufficiently high to achieve the intended benefit”. Then, they were asked to rate on a 10-point scale (1: not important; 10: very desirable) the importance of FFs against a list of different age-related health problems such as thew following: diabetes, overweight, hypercholesterolemia, high blood pressure, bowel disorders, digestive disorders, anemia, loss of strength, memory decline, pain, inflammations, anxiety/agitation, insomnia, impaired vision, loss of hearing, low mood.

#### 2.2.4. Appreciation of Possible Functional Food 

In this section, participants were introduced to the FFs co-creation task (Section 5); thus, they were asked to rate on a 10-point Likert scale (1: very unpleasant; 10: very appreciated) their appreciation of 12 possible FFs (pasta, cheese, vegetables, fruit, legumes, bread, beverage, yogurt, tea, meat, sweet, ice-cream).

#### 2.2.5. Co-Creation Task: Sensory Attributes Selection for an Acceptable, Functional Food

The participants were involved in the co-creation of tasty functional food by indicating which sensory attributes they would like or not in a FF. A total of 107 (21%) out of the 511 consumers taking part in the interview chose to quit before the co-creation task. The 403 individuals participating to this final part of the study (EEF, *n* = 108, EEM, *n* = 91, LEF, *n* = 116, and LEM, *n* = 88) were requested to pick the desired attributes from a list of 14 descriptors related to taste, flavor, texture, and serving temperature (warm or cold) by simply answering “yes” or “no”.

### 2.3. Statistic Data Analysis

Data analysis was performed by using R programming language ver.4.3.1 (R Core Team 2023. _R: A Language and Environment for Statistical Computing_. R Foundation for Statistical Computing, Vienna, Austria) and SensoMineR: Sensory Data Analysis for R package version 1.2.

Descriptive statistics were used for socio-demographic data.

One-way and two-way ANOVAs were performed on data related to the self-assessment of health status and to the questions in Section 2, Section 3 and Section 4. Tukey’s post hoc test was also used to test the differences between different groups. Differences were considered significant when *p* < 0.05. 

The co-creation data were analyzed as follows. The interaction of several key factors identified in FFs sensory characteristics and the individual choice (“yes”: positive or “no”: negative for FF acceptance) for each combination of age class and gender was analyzed using frequency tables, also shown as mosaic plots. These graphics summarized the influence of interacting variables, such as the number of subjects indicating a positive/negative impact on acceptance for each sensory characteristic. In each plot, the size of any cell represents the frequency of the subjects given the factors listed around the edges of the plot. The boundary line of the cells indicates whether the cell is larger (solid line) or smaller (dashed line) than the frequency expected if the factors were independent. The size of the residuals determines the tone of a cell: dark color for large residuals (>4), light color for medium sized residuals (<4 and >2), white color for small residuals (<2). Cut-offs are usually chosen as 2 and 4 because Pearson residuals are normally distributed, which implies that cells with these residuals are individually significant at approximately α = 0.05 and α = 0.0001 levels. The overall *p*-value was calculated from an χ2 distribution with the appropriate number of degrees of freedom. To define the association between the combination of group age and gender, sensory characteristics, and positive/negative judgement, we used multiple correspondence analysis (MCA), a method to create new perspectives (dimensions) upon the initial data.

## 3. Results

### 3.1. Socio-Demographic and Health Status Self-Assessment

In total, 511 subjects answered the questionnaire. The 46.8% aged 65 ≤ 74 were classified as early elderly (EE) and included 130 women (F) and 109 men (M), while the 53.2% aged ≥ 75 were classified as late elderly (LE), including 157 women (F) and 115 men (M) (Table 1). The sample was also divided into the three conventional Italian macro regions, with the majority, 48%, living in the northern regions, 21%, the central area, and 31% in south and islands. The percentages are in line with the Italian population distribution in 2019 [18]. The education level showed that 37.0% completed primary school, 41.1% high school, while 21.9% obtained a degree. Questions about residence zone indicated a population living in urban (48.5%) and sub-urban zones (26.8%).

Health status self-assessment and dietary habits (Table 2) indicated good diet healthiness (7.7) and health status (7.1), with no differences among predefined classes, whereas the exercise routine recorded a relatively lower score (6.1) with significant differences between females and males, regardless of the age class, with men being more active.

### 3.2. Foods Frequency Consumption and Healthiness Assessment of Foods and Food Ingredients

The participants’ daily diet was mainly based on fruits, vegetables, bread, and pasta, with a significant use of extra virgin olive oil (Table 3). Lower frequencies, but still over three (“sometimes”), were recorded for legumes, white meat, and cheese. Differences among gender and age were observed for vegetable consumption, with a higher consumption recorded for females, particularly EEF, eating more vegetables than EEM. A different behavior was observed for pasta: males, particularly LEM, declared higher pasta consumption than EEF. Differences were also observed for yogurt, which was more consumed by EEF than EEM; for EVOO, which was more used by females; and for wine, which was frequently used by males, particularly for LEM. Spices were more used by females, with a decrease with advanced age, and the lowest consumption was recorded for LEM.

The evaluation of food healthiness showed that EVOO, fruit, vegetables, and legumes were considered the healthiest foods, particularly by females compared to males (Table 4). Vegetables and legumes showed a decrease in advanced age, particularly in LEM. Seafood also received a high score (average 8.8), although this was significantly lower in LEM. High scores were also recorded for bread and pasta (7.6 and 7.7, respectively). Considerable differences were observed between red/cured meat and white meat (5.4 and 7.1, respectively), with the lowest score for red/cured meat registered by EEF, and the lowest for poultry meat registered by LEM. Yogurt, eggs, and tea were considered quite healthy (7.5, 7.1 and 7.0 scores, respectively), particularly by females compared to LEM. Cheese was considered averagely healthy (6.8), with no differences among gender and age. Wine was healthier for males, particularly LEM. Spices, sweets, and butter (5.9, 5.3, 4.7 scores) were under the threshold of “neither healthy nor unhealthy”, regardless of EEF high score for spices.

**Figure 1:**
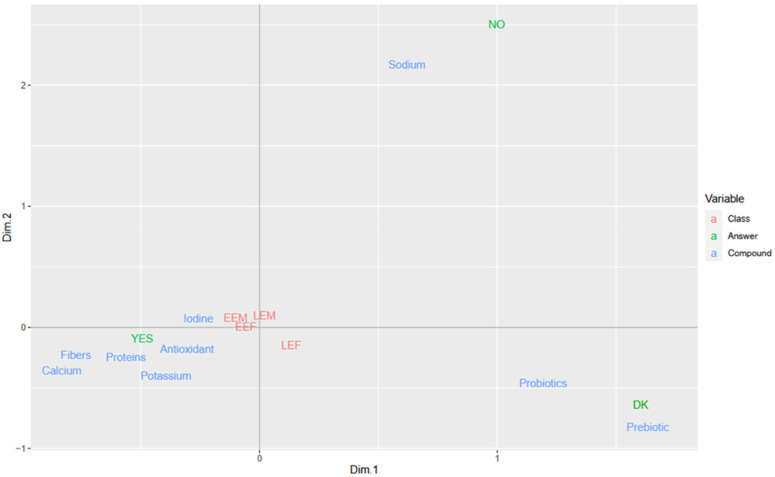
MCA plot of variables on expected impact of selected bioactive food ingredients on health.

When presented with a list of bioactive food components, the participants associated calcium, fibers, iodine, proteins, potassium, and antioxidants to a positive impact (YES) on health (Figure 1, MCA plot), sodium to a negative one (NO), while prebiotics and probiotics resulted not well known (DK). No differences were recorded among age-gender classes.

### 3.3. Expectations about Functional Foods Healthy Benefits

The participants showed trust in FFs’ healthy benefits against age-related problems. FFs were considered particularly effective against diabetes (8.9/10 score) and overweight (8.8 score), especially by women, particularly EEF compared to LEM. The same results were recorded for hypercholesterolemia, hypertensions, and bowel disorders (Table 5). As related to digestive disorders, once more, women showed higher trust in FFs, while there were no differences among age classes. Trust in FFs against anemia and strength loss was quite high (8.6 score), with women being more involved. Similar results were registered for memory decline (8.3 score), with women being more interested than men (8.5 vs. 8.0), and for pain relief (female 8.0 vs. male 7.5), anxiety/agitation (8.2 vs. 7.6), impaired vision (8.3 vs. 7.6), and insomnia (8.4 vs. 7.7). Memory decline and loss of hearing showed no difference among gender, age, and gender x age interaction. FFs were considered significantly effective against low mood (8.7/10 score), with women (9.0) being more confident than men (8.4) (Table 5).

### 3.4. Appreciation of Possible Functional Food

The potential FFs identified to be the most acceptable were fruit, vegetables, legumes, bread, and pasta (8.7, 8.4, 7.8, 7.3, 7.3, scores, respectively). Only legumes recorded differences among age classes, with lower appeal for LE classes (Table 6). Other foods were considered to be less interesting.

According to the gender differences, tea was more appreciated by females, particularly for EEF than LEM. Functional sweets was also more appreciated by females, while yogurt was less interesting for LEM.

### 3.5. Co-Creation Task: Sensory Attributes Selection for an Acceptable, Functional Food

**Figure 2:**
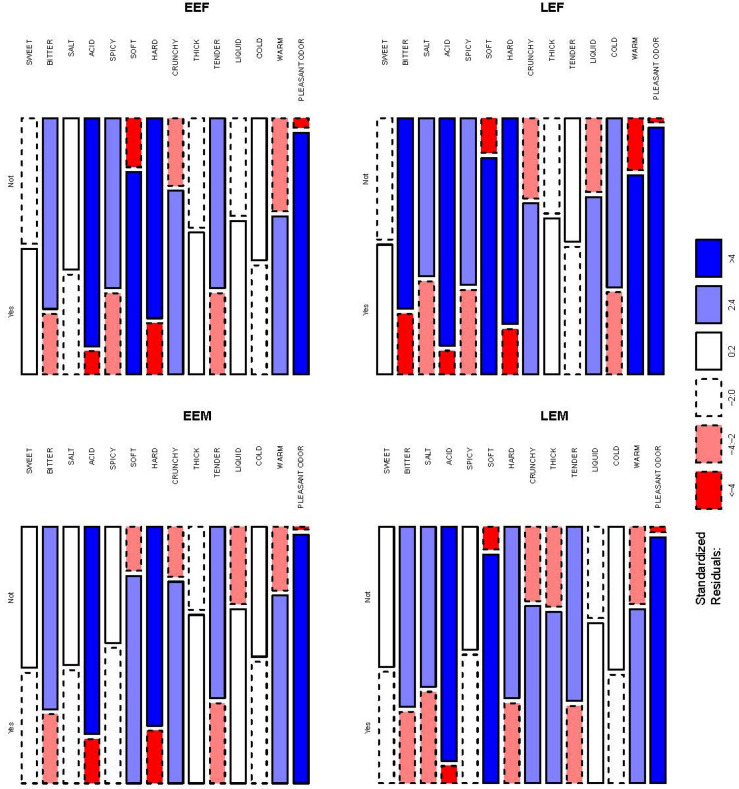
Mosaic plots of sensory characteristics for each group (EEF, LEF, EEM, and LEM). The area of each rectangle is proportional to the cell frequency of the corresponding contingency table. Solid and dashed lines indicate, respectively, positive and negative deviations from the expected frequencies. The shading of each rectangle is proportional to standardized residuals from the fitted model (values indicated in the legend). Dark and light color rectangles indicate significant deviations from the expected cell frequencies (at α = 0.05 and α = 0.0001, respectively).

The old consumers created their ideal FF by selecting the sensory attributes they would appreciate the most. There were significant differences in the frequencies of subjects indicating yes/no for each sensory attribute in each group. The groupings of blue cells with solid boundary lines in Figure 2 show clear clusters where certain sensory characteristics were overrepresented within all four groups. A cluster composed by attributes which yielded more than expected in yes: “Pleasant odor” (EEF, N = 104/108, 96%; EEM, N = 90/91, 99%; LEF, N = 114/116, 98%, LEM, N = 86/88, 98%), “soft” (EEF, N = 87/108, 81%; EEM, N = 75/91, 82%; LEF, N = 100/116, 86%, LEM, N = 80/88, 91%), and “crunchy” (EEF, N = 79/108, 73%; EEM, N = 73/91, 80%; LEF, N = 79/116, 68%, LEM, N = 62/88, 70%). A second one composed by attributes which yielded more than expected in no: “bitter” (EEF, N = 82/108, 76%; EEM, N = 66/91, 73%; LEF, N = 88/116, 76%, LEM, N = 63/88, 72%), “acid” (EEF, N = 98/108, 91%; EEM, N = 75/91, 82%; LEF, N = 105/116, 91%, LEM, N = 82/88, 93%), and “hard” (EEF, N = 86/108, 80%; EEM, N = 72/91, 79%; LEF, N = 95/116, 82%, LEM, N = 60/88, 68%). A different behavior in the four groups was observed for “salty” and “spicy” attributes. While for Late Elderly groups (LEF and LEM) “salty” yielded more than expected in no (LEF, N = 73/116, 63%, LEM, N = 56/88, 64%), for the Early Elderly groups (EEF and EEM) the “salty” attribute showed no significant differences. For the “spicy” attribute in the EEF and LEF groups, “spicy” yielded more than expected in no (EEF, N = 73/108, 68%; LEF, N = 77/116, 66%), while in the EEM and LEM groups no significant differences were found (EEM, N = 42/91, 46%; LEM, N = 43/88, 49%).

**Figure 3:**
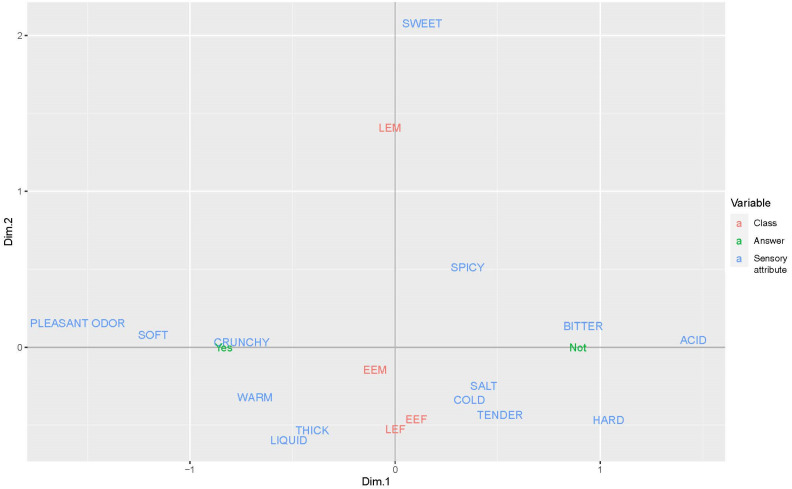
MCA plot of variables on co-creation of the ideal functional food.

The age/gender interaction analysis confirmed that pleasant odor/flavor was the most desirable trait for all four classes, and acid the least appreciated. On the other hand, salty was about neutral for EE classes, while it was excluded by LEs, particularly by LEM. Spicy was about neutral for males and was considered negative for females. Warm and liquid textures were appreciated more by LEF. Meanwhile, the least desired attributes were acid, bitter, and hard texture. Salty and soft were also not so desirable, while cold, spicy, and sweet were appreciated on average. The results based on the MCA confirm a preference for pleasant odor/flavor, soft, warm, liquid, and thick food (Figure 3).

## 4. Discussion

This study aimed to assess the Italian ageing population’s adherence to a healthy diet and their awareness of beneficial food properties by studying their attitude toward the adoption of tailored functional foods to prevent or slow down age-related disorders. The participants, despite gender and age, provided positive self-assessments of both health status and personal diet healthiness. Males declared a more regular exercise routine. These results are in line with previous studies conducted by Gall et al., 2019 [19], in southern Italy and Lee, 2005 [20], who showed that women were generally less active but more involved in household activities.

The diet composition of the interviewed population showed a significant adherence to the Mediterranean Diet (MD) principles [21,22], mainly based on fruits, vegetables, bread, pasta, and extra virgin olive oil. These are encouraging findings for elderly health perspectives, since the MD is “a model of healthy eating, because of its contribution to the state of health and its positive influence on the quality of life”. The MD represents one of the most balanced diets, that, improved by traditional products, generates considerable benefits to human longevity and well-being [23,24,25,26,27]. Specifically, the declared daily use of extra virgin olive oil (EVOO) would surely provide precious advantages against age-related problems. Indeed, the use of this vegetal fat is extremely more frequent than animal fat, the most common of which in Italy is butter, which is unhealthy because of its cholesterol-raising factor [28] and its saturated fat content. Furthermore, several researchers have highlighted the beneficial role of EVOO in counteracting age-related impairments, cognitive loss included [29,30]. Recently, D’andrea et al., 2020 [31], have shown that EVOO bioactive substances, such as hydroxytyrosol, are anti-ageing compounds having antioxidant, anti-inflammatory, and pro-apoptotic activity. Moreover, the consumption of green leafy vegetables and berry fruits containing polyphenolic compounds with anti-inflammatory and antioxidant properties can prevent or reduce the risks of cognitive decline and diseases linked with natural ageing [32,33,34,35]. Therefore, even if the MD was generally observed, some differences were found among genders, as females showed higher vegetable and EVOO consumption, while males higher pasta and wine. These findings are in line with previous research [22] and suggest that Italian males have a more traditional approach to diet than females. Males also considered wine healthier, especially at an advanced age, probably due to the popular belief t “Il vino fa buon sangue” (wine makes your blood good) rather than due to an awareness of wine’s potential benefits against coronary heart diseases, although with moderate consumption [36,37]. On the other hand, females showed a higher interest in yogurt consumption, also indicated as a beneficial food [38], and in herbs and spices, maybe due to females’ higher cooking/food skills [39]. Females, particularly those between 65 and 74 years old, showed higher awareness of some key food healthy properties such as EVOO, fruit, vegetables, and legumes, but also the negative effects of red/cured meat. Even though society’s roles are constantly challenged and changing, elderly women’s behavior is still influenced by past-century roles and responsibilities such as purchasing food, supporting family welfare, and taking care of the house [40], and this seems to provide them with a clearer consciousness about the relevance of food quality and diet in preserving well-being. On the other hand, participants, regardless of their gender, correctly indicated the potential positive and negative impacts on of selected food components health, while they were found to be not informed about prebiotics and probiotics function, suggesting the need to increase recommendations and ease the communication about functional properties of these bioactive substances [41].

Regarding the inclination toward FFs, the results indicate a clear interest in adopting them against age-related health problems. The elderly considered FFs to be effective, especially on issues correlated to eating habits, such as diabetes, fast-growing disease all over the world, and obesity, a diabetes onset factor [42].

Interestingly, the elderly had high expectations of FFs fighting low mood; indeed, late-life mood disorders increase with age and are leading public health problems [43]. Females had more trust in FFs’ efficacy against most of the selected health problems. This gender-related attitude was also observed by Welk et al., 2023 [44], in German consumers, about iron-enriched FFs used to treat fatigue and anemia.

Target consumers’ involvement in FF development provides useful information to increase products’ acceptance [45]. Thus, it was helpful to obtain indications about food categories which would give rise to well-accepted FFs and also to define specific favorite sensory traits, since they are the most important factors for a functional products’ success [46,47].

The most appealing FFs to-be were also the most frequently consumed foods, probably due to the familiarity effect acting against novel food neophobia [48]. Indeed, fruit, vegetables, and legumes were the most desirable FFs, thus, providing a chance to improve fortified and enriched fresh foods as enjoyable FFs [44]. Bread and pasta, key mediterranean foods, were also among the most cited FFs to-be, giving the FF baking industry a hint [49]. Specific sensory traits may increase the FFs appeal [46,47], and the attributes indicated by older consumers as favorites suggest that potential FFs should be characterized by a pleasant odor and flavor, softness, warmth, liquidity, and a thick texture. This information can support tailor-made FF design. However, as related to odor, in 2016 Doets and Kremer [50] highlighted that when additional pleasant flavors were added to counteract the age-related loss of acuity, no increasing liking was recorded. On the other hand, the elderly appeared to maintain better a capacity of perceiving disturbing odors. The results show sensory attributes to avoid, such as acidity, bitterness, and hard textures, providing additional indications toward the creation of adequate FFs for elderly people.

Possible limitations in this study are related to the number of participants; indeed, although the number of participants was adequate and representative for the study, according to the sample size calculator [51], a higher number of participants would have allowed for additional segmentations of the population. Moreover, although the CATI method was adequate for contacting older people, it was a time-consuming method for both the interviewer that had to second check consumers’ question understanding and for the consumers, with about 20% quitting before the end of the questionnaire. 

This study assessed the elderly’s food preferences and the ideal sensory features for creating successful FFs by considering the influence of different factors such as gender and age class. These findings give a hint to the food industry and suggest how to overcome the social and economic barriers hindering the adoption of an adequate diet and an active lifestyle at advanced age [52]. In addition, since at an advanced age neophobic behaviors may arise [53], education programs and engagement strategies can play an important role in increasing knowledge and trust in functional foods and also keeping the motivational issue of food curiosity alive at late age.

## 5. Conclusions

The elderly Italian population participating in our study showed a consistent adoption of healthy foods and an encouraging awareness about food healthiness, especially in females. Interestingly, the elderly showed interest and trust in foods able to provide health benefits, suggesting opportunities for increasing the successful consumption of tailor-made FFs by ageing people. The CATI interview approach was effective in contacting and supporting the old participants in expressing their habits, knowledge, and expectations. The co-creation methodology, adopted for defining both the most suitable food matrices and the sensory profiles of an acceptable FF to-be, was an appropriate method for involving the elderly and letting them share their preferences and dislikes. The results of this investigation can be helpful for understanding the elderly’s attitudes toward healthy and tasty functional foods, but also in the healthcare sector to better promote the beneficial effect of functional foods, as well as for food industry stakeholders to develop innovative products based on the elderly’s tastes and preferences. Multi-disciplinary studies, including medical, nutritional, and sensory approaches, would be effective in promoting health-enhancing foods for the elderly but require the support of an effective communication which is necessary for overcoming age-related prejudices and restraints slowing down beneficial food changes.

## Figures and Tables

**Table 1:** Socio-demographic characteristics of the sample population.

	65–74		≥75		TOT
	Early Elderly (*n* = 239) 46.8%		Late Elderly (*n* = 272) 53.2%		(*n* = 511)
	Female (*n* = 130)	Male (*n* = 109)	Female (*n* = 157)	Male (*n* = 115)	
**Gender and Age**	25.4%	21.3%	30.7%	22.5%	100%
**Geographical areas (Italy)**					
North	40.8%	39.4%	55.4%	55.7%	48.3%
Central	20.0%	23.9%	20.4%	19.1%	20.7%
South and Islands	39.2%	36.7%	24.2%	25.2%	30.9%
**Level of education**					
Primary school	33.8%	29.4%	45.2%	36.5%	37.0%
High school	41.5%	43.1%	42.0%	37.4%	41.1%
University	24.6%	27.5%	12.7%	26.1%	21.9%
**Residence**					
Urban	45.4%	49.5%	45.9%	54.8%	48.5%
Suburb	27.7%	24.8%	28.7%	25.2%	26.8%
Rural	19.2%	22.0%	17.2%	16.5%	18.6%
Other (town, mountain, seaside)	7.7%	3.7%	8.3%	3.5%	6.1%

**Table 2:** Participants self-assessment of diet healthiness, health status, and exercise routine. Statistical differences were determined by one-way ANOVA using “gender & age groups” factor, and two-way ANOVA using age, gender, and their interaction. Different letters (a, b, c) correspond to significantly different means according to Tukey post hoc test (*** *p* < 0.001).

	Healthy Diet	Health Status	Exercise Routine
**Total**	7.73	7.13	6.08
**Gender and Age groups**			
EEF	7.73	7.26	5.73 ^bc^
LEF	7.64	7	5.35 ^c^
EEM	7.82	7.06	6.81 ^a^
LEM	7.73	7.21	6.44 ^ab^
Age			
Gender			***
Age × Gender			

**Table 3:** Frequency consumption of 17 common food categories, assessed on a 5-point scale: 1 = “never”; 2 = “rarely”; 3 = “sometimes”; 4 = “often”; 5 = “everyday”. Statistical differences were determined by one-way ANOVA using “gender & age groups” factor, and two-way ANOVA using age, gender, and their interaction. Different letters (a, b, c) correspond to significantly different means according to Tukey post hoc test (* *p* < 0.05; ** *p* < 0.01; *** *p* < 0.001).

Gender and Age Groups	FOOD Category																
	Fruit	Vegetable	Legumes	Bread	Pasta	Sweets	Red/Cured Meat	White Meat	Sea Food	Eggs	Cheese	Butter	Yogurt	Evoo	Wine	Tea	Spices
*p*		**			**												
EEF	4.68	4.63 a	3.35	4.49	3.9 b	3.05	2.88	3.35	3.26	2.94	3.33	2.01	3.03 a	4.93 a	2.56 c	3.00	3.26 a
LEF	4.76	4.40 ab	3.26	4.38	4.01 ab	3.21	2.95	3.34	3.11	2.88	3.66	2.20	2.63 ab	4.82 a	2.85 bc	2.85	3.04 ab
EEM	4.73	4.24 b	3.47	4.51	4.19 ab	2.96	3.10	3.42	3.36	2.91	3.62	2.00	2.54 b	4.61 b	3.27 ab	2.58	3.07 ab
LEM	4.78	4.36 ab	3.27	4.53	4.30 a	2.92	2.99	3.41	3.12	2.94	3.70	2.00	2.57 ab	4.76 ab	3.78 a	2.59	2.74 b
Age									*		*				*		*
Gender		**			***								*	**	***	*	*
Age × Gender		*												*			

**Table 4:** Food healthiness evaluation of 17 common food categories assessed on a 10-point scale: 1 = “not healthy at all”; 6 = ”neither healthy nor unhealthy”, 10 = “extremely healthy”. Statistical differences were determined by one-way ANOVA using “gender & age groups” factor, and two-way ANOVA using age, gender, and their interaction. Different letters (a, b, c) correspond to significantly different means according to Tukey post hoc test (* *p* < 0.05; ** *p* < 0.01; *** *p* < 0.001).

Gender and Age Groups	FOOD Category																										
	Fruit	Vegetables	Legumes		Bread	Pasta	Sweets	Red/Cured Meat		White Meat		Seafood		Eggs		Cheese	Butter	Yogurt		Evoo		Wine		Tea		Spices	
*p*	***	***	***					**		**		***		**				***		***		*		**		**	
EEF	9.36 a	9.51 a	8.97 a		7.37	7.82	5.07	4.83 b		7.40 ab		9.06 a		7.46 a		6.73	4.57	7.85 a		9.45 a		5.73 b		7.57 a		6.36 a	
LEF	9.24 a	9.22 ab	8.51 b		7.79	7.71	5.68	5.74 a		7.91 a		8.87 a		7.30 a		6.96	5.05	7.98 a		9.48 a		5.97 ab		7.12 ab		5.91 ab	
EEM	8.99 ab	9.05 b	8.60 ab		7.49	7.61	5.33	5.53 a		7.58 ab		8.94 a		6.98 ab		6.64	4.86	7.50 ab		9.03 b		6.11 ab		7.07 ab		5.92 ab	
LEM	8.70 b	8.61 c	7.76 c		7.57	7.79	5.28	5.65 a		7.14 b		8.18 b		6.73 b		6.71	4.51	6.79 b		8.94 b		6.73 a		6.36 b		5.21 b	
Age		**	***					**				***												*		*	
Gender	***	***	***							*		***		***				***		***		**		**		**	
Age × Gender								*		**		*						*									

**Table 5:** Health problems which participants would be willing to counteract with functional foods. (1: not important; 10: extremely desirable). Statistical differences were determined by one-way ANOVA using “gender & age groups” factor, and two-way ANOVA using age, gender, and their interaction. Different letters (a, b, c) correspond to significantly different means according to Tukey post hoc test (* *p* < 0.05; ** *p* < 0.01; *** *p* < 0.001).

Gender and Age Groups	Diabetes	Overweight	Hypercholesterolemia	Hypertension	Bowel Disorders	Digestive Disorders	Anemia	Loss of Strenght	Memory Decline	Soreness	Inflamations	Anxiety Agitation	Insomnia	Impaired Vision	Loss of Hearing	Low Mood
*p*	**	**	*	**	**		***	*		*		**	**	*		**
EEF	9.19 a	9.11 a	8.93 a	8.77 a	9.09 a	8.71	8.82 ab	8.80 a	8.64	7.91 ab	7.94	8.11 ab	8.25 ab	8.24 ab	7.42	8.87 ab
LEF	9.00 ab	8.87 a	8.72 ab	8.88 a	8.81 ab	8.8	9.06 a	8.74 ab	8.49	8.17 a	8.26	8.36 a	8.53 a	8.35 a	7.62	9.08 a
EEM	8.72 ab	8.68 ab	8.66 ab	8.54 ab	8.51 b	8.41	8.42 bc	8.27 b	7.97	7.27 b	7.62	7.60 b	7.66 b	7.55 b	7.01	8.34 b
LEM	8.51 b	8.27 b	8.33 b	8.16 b	8.49 b	8.36	8.07 c	8.35 ab	8.08	7.64 ab	7.86	7.64 b	7.70 b	7.81 ab	7.35	8.51 b
Age																
Gender	**	**	*	**	***	**	***	**	**	**		***	***	**		***
Age × Gender							*									

**Table 6:** Potential FFs preferred by participants. Scale 1–10 (1: not used in any case; 10: would use very gladly). Statistical differences were determined by one-way ANOVA using the “gender & age groups” factor, and two-way ANOVA using age, gender, and their interaction. Different letters (a, b) correspond to significantly different means according to Tukey post hoc test (* *p* < 0.05; ** *p* < 0.01).

Gender and Age Groups	Food Category											
	Fruit	Vegetables	Legumes	Bread	Pasta	Sweet	Ice Cream	Meat	Cheese	Yogurt	Drink	Tea
*p*						*						
EEF	8.91	8.66	8.11	7.22	7.31	6.27 a	6.33	5.52	6.51	6.87 a	6.02	6.41
LEF	8.66	8.39	7.52	7.09	7.20	6.01 ab	6.12	6.05	6.40	6.27 ab	6.21	6.20
EEM	8.59	8.22	8.05	7.25	7.50	5.93 ab	6.37	6.12	6.62	6.35 ab	6.34	5.71
LEM	8.51	8.17	7.56	7.49	7.33	5.21 b	5.55	6.10	6.61	5.84 b	6.63	5.77
Age			**							*		
Gender						*						*
Age × Gender												

## Data Availability

The data presented in this study are available on request from the corresponding author. The data are not publicly available due to data privacy.
